# End-to-end molecular structure elucidation from multimodal NMR spectra images using vision transformers

**DOI:** 10.1039/d6sc02352e

**Published:** 2026-05-26

**Authors:** Chao Han, Xiaolin Pan, Yingkai Zhang

**Affiliations:** a Department of Chemistry, New York University New York 10003 USA yingkai.zhang@nyu.edu; b Simons Center for Computational Physical Chemistry at New York University New York New York 10003 USA; c NYU-ECNU Center for Computational Chemistry at NYU Shanghai Shanghai 200062 China

## Abstract

Nuclear magnetic resonance (NMR) spectroscopy is a cornerstone technique for molecular structure elucidation, but interpreting complex NMR spectra remains challenging and often relies on expert-driven, heuristic workflows. Recent deep learning approaches have enabled spectrum-to-structure prediction, yet many depend on peak-level text annotations that discard informative intensity patterns and are not readily extended to higher-dimensional NMR experiments. Here, we present NMRViT, a spectral Vision Transformer framework for NMR-driven molecular structure elucidation that operates directly on raw spectral signals of both one-dimensional (^1^H, ^13^C) and two-dimensional (HSQC) spectra. Trained on a large-scale simulated dataset, NMRViT achieves strong performance across single-modality and multimodal NMR spectral input configurations, and provides, to our knowledge, one of the first end-to-end benchmarks for HSQC-based structure prediction. We systematically evaluate both zero-shot transfer and fine-tuning from large-scale simulated data on two experimental benchmarks covering 1D and 2D NMR spectra, highlighting the simulation–experiment domain gap and showing that fine-tuning with only a small number of experimental samples can substantially reduce this gap. To further improve candidate ranking, we introduce a chemical-shift-based post-processing strategy that re-ranks candidate structures using local spectral evidence, yielding consistent performance gains in both zero-shot and fine-tuning settings. Together, these results establish raw-spectrum vision transformers, combined with lightweight experimental adaptation and chemically informed re-ranking, as a practical framework for automated molecular structure elucidation from multimodal NMR data.

## Abstract 2

NMRViT is an end-to-end vision transformer that learns directly from raw ^1^H, ^13^C and HSQC NMR spectra to predict molecular structures, advancing automated structure elucidation from multimodal spectral data.

## Graphical abstract

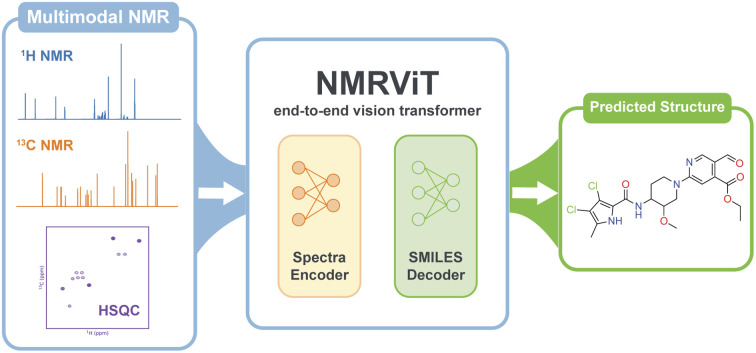

## Introduction

Nuclear magnetic resonance (NMR) spectroscopy is a central analytical technique to probe molecular structures and dynamics.^1–4^ Despite its broad applicability, determining a complete molecular structure directly from NMR spectra remains challenging in practice and often relies on expert-driven interpretation strategies that combine multiple spectral features and heuristic reasoning. This process can be time-consuming and may become particularly difficult for structurally complex molecules or when spectral overlap and experimental noise obscure key signals.

Computational methods have long been developed to facilitate NMR spectral interpretation by comparing predicted spectra with experimental measurements to identify the most consistent structures among candidate molecules. These spectrum prediction approaches include both quantum-chemical calculations, such as density functional theory (DFT), and data-driven machine learning models.^5–17^ While effective in many applications, spectra prediction approaches generally rely on a predefined pool of candidate structures and therefore cannot directly generate or extrapolate to previously unseen molecules. In addition, most computational pipelines treat individual NMR experiments separately, whereas jointly interpreting complementary spectral modalities can substantially improve structural resolution but remains difficult to perform in a systematic and automated manner.

Recent advances in deep learning have shifted spectrum-based structure elucidation from candidate scoring and retrieval toward end-to-end prediction, enabling models to directly map spectral inputs to molecular structures.^18–35^ For example, Alberts *et al.*^20^ introduced a large simulated multimodal spectroscopic dataset with accompanying benchmarks, and the following work^21^ further developed a multitask transformer on this dataset that integrates NMR and Infrared (IR) information. Their works and many other transformer-based works encode spectra *via* extensive preprocessing into texts like peak lists and multiplicity symbols, which may discard informative intensity patterns and introduce heuristic biases. In parallel, emerging studies^18,36–38^ have explored predicting molecular structures directly from minimally processed spectral signals and reported encouraging results for one-dimensional NMR inputs. This progression motivates further investigation into whether raw-spectra-based models can scale to structurally more complex molecules and effectively integrate multiple complementary NMR modalities, including both one-dimensional (1D) and two-dimensional (2D) experiments. In particular, 2D HSQC spectroscopy provides direct correlations between ^1^H and directly bonded ^13^C nuclei, offering connectivity constraints that can help resolve structural ambiguities that are difficult to address from 1D spectra alone.^39–41^ Alongside ongoing architectural exploration, improving the transferability of spectrum-to-structure models from simulated to experimental data has emerged as another important challenge. Models developed on simulated datasets frequently show degraded performance when transferred to experimental spectra,^18,21^ making it essential to address this simulation–experiment domain gap for practical usage in real NMR-based structure elucidation.

In this work, we present NMRViT, an end-to-end spectral Vision Transformer framework for NMR-driven molecular structure elucidation that directly operates on raw NMR spectral signals. Our model ingests multiple NMR modalities including 1D ^1^H, 1D ^13^C, and 2D HSQC spectra and predicts molecular structures in a unified sequence-generation setting. We further incorporate a molecular-formula prompt as auxiliary conditioning information and employ patch dropout as a regularization strategy to improve robustness. Trained on a large-scale simulated dataset, NMRViT achieves competitive performance relative to previously reported spectrum-to-structure models and establishes a strong baseline for HSQC-based structure prediction. We further evaluate model generalization on experimental benchmarks, including the Chemical Education dataset for 1D spectra and the HMDB-HSQC dataset for 2D HSQC measurements. Zero-shot transfer reveals a clear simulation–experiment performance gap, whereas fine-tuning on small experimental subsets substantially narrows this gap and enables competitive structure prediction accuracy. In addition, we introduce a chemical-shift-based re-ranking strategy that refines generated candidates using local spectral evidence, leading to consistent performance improvements in both zero-shot and fine-tuned settings. Overall, these results demonstrate the effectiveness of NMRViT models combined with lightweight experimental adaptation for advancing automated molecular structure elucidation from multimodal NMR data.

## Methods

### Data set processing

#### Simulated multimodal spectroscopic dataset

**Fig. 1:**
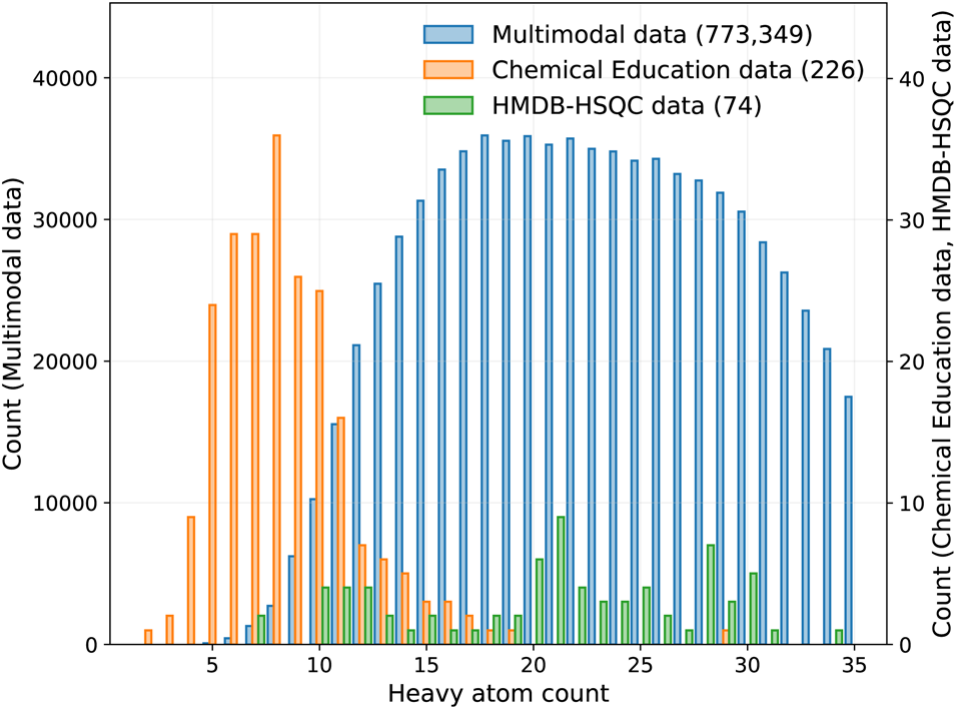
Distribution of molecular size across the datasets used in this work. Histograms show the number of molecules as a function of heavy-atom count for the large-scale simulated multimodal dataset (blue), the experimental Chemical Education dataset (orange), and the experimental HMDB-HSQC dataset (green). The simulated dataset covers a broad range of molecular sizes (5–35 heavy atoms). The Chemical Education dataset provides a fundamental evaluation across a diverse set of relatively small organic molecules, whereas the HMDB-HSQC dataset offers a more challenging test with fewer but generally larger and more structurally complex structures.

We primarily train and evaluate our models using the large-scale simulated Multimodal Spectroscopic Dataset introduced by Alberts *et al.*,^20^ which comprises approximately 790k unique organic molecules extracted from the USPTO patent reaction corpus.^42^ Molecules are filtered to contain 5–35 heavy atoms and common organic elements (C, H, O, N, S, P, Si, B, and halogens), yielding a chemically realistic distribution representative of synthetic chemistry. For each molecule, multiple spectroscopic modalities are simulated, including 1D ^1^H NMR, 1D ^13^C NMR, 2D HSQC NMR, IR spectra, and MS/MS spectra. NMR spectra are generated using MestReNova^43^ under standardized conditions (CDCl_3_ solvent, ^13^C spectra proton-decoupled), with 1D NMR represented as dense vectors of length 10 000 and HSQC spectra represented as 2D matrices. The dataset provides both raw spectral arrays and peak-level annotations, alongside SMILES strings and molecular formulas, enabling flexible use across spectrum-to-structure, spectrum-to-property, and multimodal learning tasks (Fig. 1).

To ensure consistency and robustness across heterogeneous NMR data sources, we apply additional preprocessing to adapt the simulation dataset to our model. First, because NMR spectra from different simulators, instruments, and processing pipelines use incompatible intensity scales, raw peak intensities can carry different physical meanings across datasets, hindering transferability. We therefore perform instance-wise normalization for both 1D and 2D NMR spectra, rescaling each spectrum independently such that intensities lie in the range [0, 1]. This normalization removes dataset-specific scaling factors and facilitates stable training as well as seamless transfer from simulated spectra to experimental data. Second, we observe that molecular structures in the source dataset inconsistently encode stereochemistry: some molecules include stereochemical annotations in SMILES, while others with potential stereocenters do not, reflecting heterogeneity in upstream USPTO curation. To avoid introducing ambiguous supervision signals, we convert the prediction target to non-stereochemical SMILES and remove duplicate entries that collapse to the same canonical SMILES after stereochemistry is stripped, ensuring a one-to-one mapping between spectra and structural targets.

#### Experimental chemical education dataset

To evaluate model performance on real-world NMR data, we use the experimental spectroscopy dataset curated by Van Bramer and Bastin for undergraduate chemical education.^44^ This dataset contains 251 organic compounds with a total of 2337 experimentally measured multimodal spectra. NMR spectra were acquired primarily on 400 MHz Bruker instruments, with most samples prepared in CDCl_3_ and referenced to TMS, and were manually processed and verified by the authors. To adapt this dataset to our model, we write automated MestReNova scripts to extract binned 1D ^1^H and ^13^C spectra from the original MNova workbooks and convert them into dense vector representations compatible with the simulation-based training pipeline. Reference and solvent peaks are removed using fixed chemical-shift windows, and instance-wise normalization is applied to rescale each spectrum to the [0,1] intensity range. This results in a curated experimental benchmark comprising 226 molecules with aligned ^1^H and ^13^C NMR spectra.

#### Experimental HMDB-HSQC dataset

To further assess model performance on experimentally acquired two-dimensional spectra as complementary evaluation, we constructed an HMDB-HSQC evaluation dataset from the Human Metabolome Database (HMDB).^45^ The HMDB is a comprehensive reference resource with curated information on human metabolites and their associated chemical and spectroscopic properties. We selected compounds from HMDB with available experimental HSQC peak lists recorded in CDCl_3_, consistent with the solvent conditions used in the Multimodal Spectroscopic Dataset. Molecules containing elements outside the training distribution and several exceptionally large structures were excluded, resulting in a curated set of 74 molecules. Because HMDB provides processed cross-peak lists rather than fully digitized raw spectral matrices, we reconstruct two-dimensional HSQC intensity images from reported peak positions and intensities to serve as model inputs. A mild Gaussian broadening was applied to ^1^H dimension to better approximate the appearance of continuous HSQC spectra. The reconstruction from peak lists to HSQC spectra may omit fine spectral features, and future work will explore direct learning from raw experimental HSQC spectra when such data become available.

### Model architecture

#### Transformer for spectrum-to-structure prediction

**Fig. 2:**
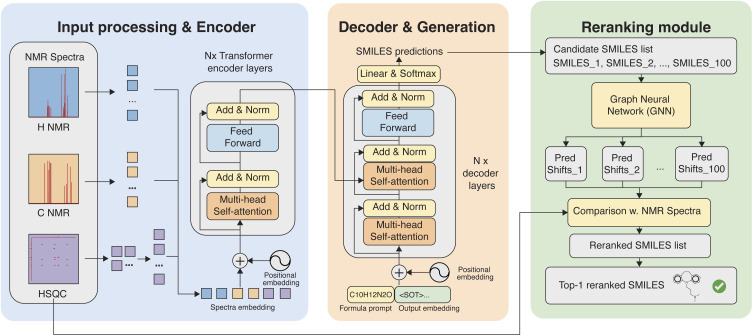
Overview of the NMRViT framework for multimodal spectrum-to-structure prediction. Raw 1D (^1^H, ^13^C) and HSQC spectra are partitioned into modality-specific patches and encoded by a shared Vision Transformer encoder. An autoregressive Transformer decoder generates candidate SMILES sequences conditioned on the encoded spectral representations and a molecular-formula prompt. Multiple candidates produced by beam search are subsequently refined using a chemical-shift-based re-ranking module driven by external graph neural network predictions, yielding the final top-ranked structure.

We formulate NMR-driven structure elucidation as a sequence-generation task that maps one or more NMR spectra to a molecular SMILES representation, a standardized text notation that encodes molecular structures as text strings.^46^ After preprocessing, both 1D and 2D spectra are represented as fixed-size numerical intensity fields on discrete chemical-shift grids and are used directly as model inputs. To learn the spectrum–structure relationship, we employ an encoder–decoder Transformer architecture.^47^ The spectral encoder adopts the design of the Vision Transformer (ViT),^48^ in which spectra are partitioned into local patches and embedded as tokens with positional encodings. Stacked self-attention layers then capture long-range correlations across chemical-shift regions and integrate information from multiple spectral modalities. The decoder is an autoregressive Transformer that generates SMILES tokens sequentially while attending to the encoded spectral representations through cross-attention, enabling end-to-end prediction of molecular structures from raw NMR signals (Fig. 2).

To further assist structure generation, we incorporate the molecular formula as an explicit prompt to the decoder. Concretely, we reserve a fixed prefix region at the beginning of the decoder input sequence, into which the molecular formula is written as a short sequence of tokens prior to SMILES generation. During training and inference, the decoder first consumes this molecular-formula prefix and then generates the SMILES tokens conditioned on both the encoded spectra and the formula context. This design allows subsequent SMILES tokens to attend directly to the molecular formula through self-attention, providing a global compositional constraint that helps disambiguate structures with similar spectral features but different elemental compositions. SMILES strings and molecular formulas are tokenized using a chemistry-aware regular-expression tokenizer that splits sequences into atom-level and syntax tokens, following established practice in the Molecular Transformer.^49^

In the present study, prediction targets are defined at the level of molecular constitution rather than stereochemistry. As described in the dataset processing section, all molecular structures are converted to canonical non-stereochemical SMILES during preprocessing to ensure consistent supervision. Consequently, model performance reflects the ability to recover the correct bonding topology and functional-group arrangement from spectral inputs, while rigorous inference of stereochemical configurations is beyond the scope of the current work and will be addressed in future studies.

#### Patch-based spectra encoder

We design a Vision Transformer encoder for NMR spectra by adapting the ViT framework, which represents structured inputs as sequences of embedded patches processed by a standard Transformer encoder. Both 1D and 2D NMR spectra can be viewed as intensity fields defined over discretized chemical-shift coordinates, analogous to pixel intensities on spatial grids. For 2D HSQC spectra, the input is a single-channel intensity matrix over the ^1^H and ^13^C chemical-shift axes. Each HSQC spectrum is partitioned into non-overlapping square patches, flattened, and projected into a fixed-dimensional latent space using a trainable linear layer, with learnable positional embeddings added to preserve chemical-shift information. The resulting token sequence is processed by a stack of Transformer encoder layers with multi-head self-attention, feed-forward blocks, residual connections, and layer normalization.

For 1D NMR spectra, we adopt a closely related but computationally more efficient design. Instead of reshaping 1D spectra into artificial 2D images, we partition each spectrum into contiguous 1D patches along the chemical-shift axis. Each patch is directly projected into the latent embedding space using a linear layer, without an explicit flattening step. This approach preserves the native one-dimensional structure of the data while substantially reducing the number of tokens required for a given spectral resolution. Compared to embedding 1D spectra as 2D pseudo-images, this strategy maintains higher effective resolution along the chemical-shift axis and leads to improved computational efficiency, particularly for long high-resolution spectra.

#### Multimodal spectra integration

To leverage complementary information from multiple NMR modalities, we adopt a shared-encoder fusion design. Each available modality is first partitioned into patches and mapped to a common latent dimension using a modality-specific linear embedding layer. After positional embeddings are added, the resulting token sequences are concatenated along the sequence dimension to form a single multimodal token stream, which is processed by one Transformer encoder shared across all modalities. The decoder attends to the full fused token sequence *via* cross-attention to generate SMILES autoregressively, identical to the single-modality setting. Compared with using separate encoders per modality, this architecture introduces only lightweight additional parameters in the modality-specific patch embedding layers while sharing the main encoder capacity. We also implemented a late-fusion multimodal variant that employs separate modality-specific encoders to assess whether stronger modality disentanglement could improve the performance. The model details and performance comparison are provided in the SI (Section S5).

### Training strategy

#### Patch dropout for sparse spectral inputs

NMR spectra are intrinsically sparse, with only a small fraction of chemical-shift regions containing informative signals and large background regions exhibiting near-zero intensity. When represented as patch tokens, this sparsity leads to many patches that carry little or no information, which can dominate the input sequence and hinder effective training by encouraging the model to over-attend to uninformative regions, slowing convergence and reducing robustness. To address this issue, we employ patch dropout, a regularization strategy inspired by prior work on vision-language pretraining and efficient Vision Transformers, in which a random subset of patch tokens is dropped during training.^50,51^ Specifically, patch dropout is applied to spectral input by randomly removing a fraction of patch embeddings before they are passed to the Transformer encoder. Patch tokens are dropped *via* uniform random sampling with a fixed dropout rate, without weighting by spectral intensity. For multimodal inputs, patch dropout is applied after modality-specific patch embeddings are concatenated into a single sequence, such that tokens are sampled jointly across all modalities rather than independently within each modality. Patch dropout forces the model to rely on diverse subsets of spectral evidence rather than memorizing fixed peak configurations, improves robustness to missing or weak signals, and acts as an effective regularizer for sparse spectral inputs. Patch dropout is disabled at inference time, where all available patches are retained.

#### Training and evaluation protocols

All models are trained primarily on the simulated dataset using a 7 : 2 : 1 split for training, validation, and testing, consistent with prior work to enable fair comparison.^21^ Training is performed on a single NVIDIA L40S GPU, where full spectrum-to-SMILES training for 200 epochs typically requires approximately 25–30 hours. Optimization is carried out using AdamW^52^ with an initial learning rate of 3 × 10^−4^, using a linear warmup over 10 000 steps followed by cosine learning-rate decay over the full training schedule. Other training details and hyperparameter choices are summarized in the SI (Section S1, S2).

During evaluation, generated SMILES strings are first checked for chemical validity using RDKit.^53^ Prediction accuracy is assessed by converting both predicted and target molecules to canonical, non-stereochemical SMILES strings and comparing exact matches. At inference time, the autoregressive decoder can generate multiple candidate SMILES strings using beam search, allowing evaluation of top-*k* accuracy in addition to the top-1 prediction.

### Bridging simulation-trained models to experimental spectra

Although models trained on large-scale simulated datasets have provided strong performance for spectrum-to-structure prediction,^21,22^ their direct application to experimental measurements is limited by the simulation–experiment domain gap. In this work, we investigate two complementary strategies to improve practical performance on experimental NMR data. The first approach is fine-tuning, in which the simulation-pretrained model is further trained on a small set of experimentally acquired spectrum–structure pairs. This procedure enables the model to adapt its internal representations to experimental-specific characteristics such as baseline distortions and noise patterns, thereby improving predictive accuracy without requiring changes to the model architecture. The fine-tuning strategy is evaluated on both experimental datasets, which performance is shown in the following sections.

The second approach is prediction re-ranking, a lightweight post-processing strategy designed to enhance accuracy without retraining the main generative model. During inference, the autoregressive Transformer decoder can produce multiple candidate SMILES sequences through beam search, which maintains several high-probability decoding paths in parallel and thus yields a list of plausible molecular structures. These candidates are subsequently scored using external spectral prediction methods, allowing complementary sources of chemical information to guide the final selection.

In principle, a broad spectrum of methods ranging from high-level DFT calculations to fast machine-learning models can serve as spectral predictor, and the scoring function can be flexibly designed to reflect different notions of spectral agreement. To illustrate the effect of re-ranking in this work, we employ chemical-shift prediction models CSTShift from our previous study,^11^ which are three-dimensional graph neural networks trained on experimentally annotated 1D NMR datasets. For the present re-ranking experiments, we retrained the CSTShift model on the NMRShiftDB2-based dataset after removing molecules overlapping with the experimental benchmarks used in this work, and used MMFF-calculated geometries instead of DFT-optimized geometries to enable efficient candidate scoring without requiring DFT calculations during re-ranking. From the candidate list generated by beam search, we retain up to 100 molecules and first filter them to ensure chemical validity and consistency with the known molecular formula. For each remaining candidate structure, the CSTShift model is then used to predict both ^1^H and ^13^C atomic chemical shifts. To compare predicted shifts with experimental spectra, we introduce a local evidence score that evaluates how strongly each predicted shift is supported by nearby spectral intensity. For a normalized experimental spectrum *y*(*δ*) defined on a discrete shift grid {*δ*_*j*_}, and *N* predicted shifts {*s*_*i*_}, the score is defined as
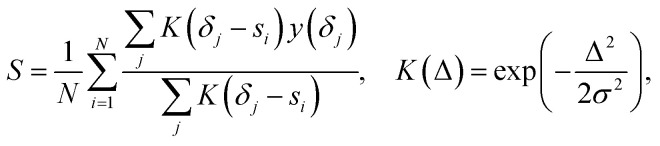
where *K*(Δ) is a Gaussian kernel with bandwidth *σ* determined by the expected chemical-shift tolerance for the corresponding nucleus. Intuitively, this metric measures the average local spectral intensity surrounding each predicted peak, thereby rewarding candidate structures whose predicted shifts coincide with experimentally supported signal regions. This formulation operates directly on spectral intensity fields without requiring explicit peak picking and can be naturally extended to higher-dimensional experiments such as 2D HSQC. To reduce the dominance of a small number of very intense HSQC cross-peaks during candidate evaluation, a nonlinear intensity rescaling with a power exponent of 0.3 is applied to compress the dynamic range of the reconstructed HSQC matrix before reranking. Further comparisons of alternative scoring strategies are provided in the SI (Section S8).

## Results and discussion

### Performance on large-scale simulation benchmark

We first evaluate our proposed end-to-end Transformer on the large-scale simulated benchmark. We compare against prior reported benchmark results by Alberts *et al.*,^21^ which employ Transformer models with the input of annotated NMR peak lists. In contrast to these approaches, our model directly consumes full spectral signals and naturally extends to two-dimensional NMR inputs. As summarized in Table 1, the proposed framework achieves competitive or superior performance across various spectral settings.

**Table 1:** Top-*k* molecular structure prediction accuracy (%) on the simulated multimodal dataset. Prior results are directly cited from the literature for reference and dashes indicate metrics not reported in the original work. Performance of the proposed NMRViT are shown for single-modality and multimodal spectral configurations

Model	Top-1	Top-5	Top-10
**Prior baseline^21^**			
^13^C NMR	49.35	66.85	—
^1^H NMR	60.08	76.74	—
^1^H + ^13^C + IR	73.40	87.83	—

**NMRViT**			
^13^C NMR	51.34	65.89	69.34
HSQC (2D NMR)	67.97	81.59	84.50
^1^H NMR	71.39	83.79	86.22
^1^H + ^13^C NMR	74.07	85.68	87.88
^1^H + ^13^C + HSQC	79.12	89.34	91.02

For single-modality inputs, the model achieves comparable accuracy to prior work on ^13^C NMR, while substantially improving performance on ^1^H NMR. In particular, the ^1^H-only model attains a top-1 accuracy of 71.39%, exceeding previously reported results that rely on peak-level annotations. This improvement is likely attributable to the ability of the raw-spectra encoder to exploit fine-grained intensity patterns that are discarded during peak picking and symbolic encoding. By contrast, ^13^C-only performance remains lower than ^1^H. Detailed analysis provided in the SI (Section S4) indicates that ^13^C spectra are significantly more sparse than ^1^H spectra, with fewer informative regions per molecule. This extreme sparsity may limit the effectiveness of patch-based representations even with patch dropout, and partially explains the observed performance gap between the two modalities.

Notably, we observe strong results for HSQC-only inputs, achieving a top-1 accuracy of 67.97%. To our knowledge, this represents one of the first systematic benchmarks for structure elucidation using 2D NMR spectra alone. These results demonstrate that the NMRViT can learn chemically meaningful correlations from 2D NMR intensity patterns and establish a competitive baseline for future work on 2D spectrum-driven structure prediction.

Combining multiple spectral modalities further improves performance but exhibits diminishing returns on the simulated dataset. Incorporating all three modalities yields the strongest overall performance, indicating that the 2D correlation information in HSQC provides additive value beyond 1D spectra alone. These results highlight that the proposed multimodal integration strategy effectively aggregates heterogeneous spectral evidence within a unified Transformer framework.

**Fig. 3:**
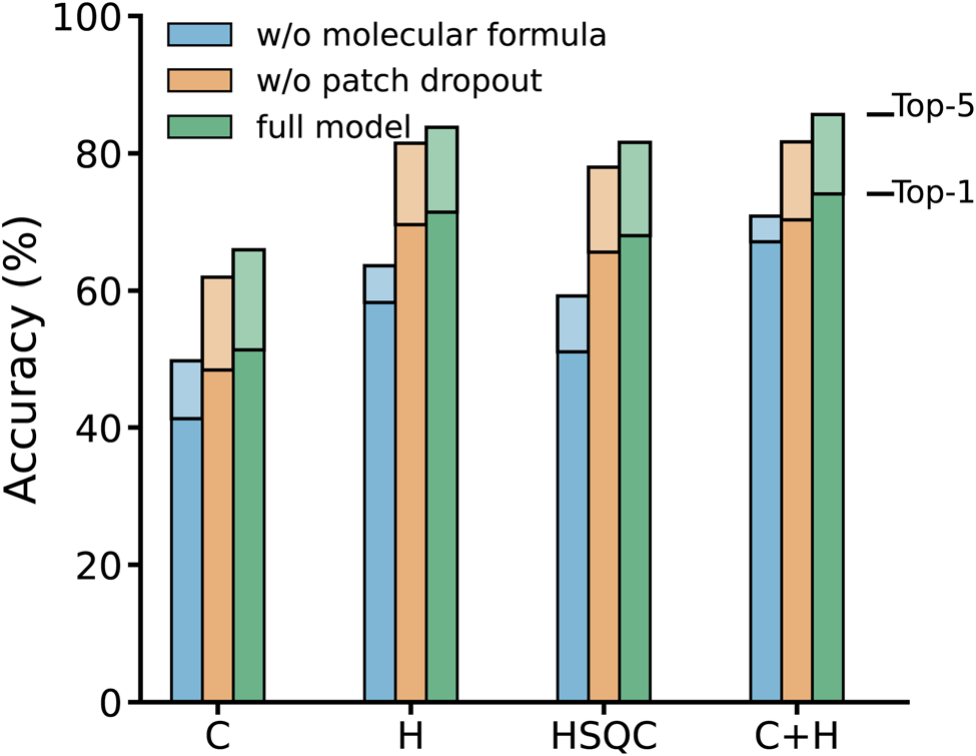
Ablation analysis of architectural components across spectral input configurations. top-1 and top-5 molecular structure prediction accuracy on the simulated benchmark are compared for models without molecular-formula prompting, without patch-dropout regularization, and the full NMRViT model. Results are shown for four spectral inputs (^13^C, ^1^H, HSQC, and ^1^H + ^13^C). Both molecular-formula conditioning and patch dropout consistently improve prediction accuracy. The full table is included in the SI (Section S3).

We further examine the performance boost with molecular formula prompt and patch dropout regularization, as evaluated in the ablation study shown in Fig. 3. Removing the molecular formula conditioning consistently reduces top-1 and top-5 accuracy across all modality settings, indicating that global compositional constraints provide important search-space regularization during autoregressive decoding. Similarly, disabling patch dropout leads to a systematic performance decline, highlighting its role as an effective regularization strategy. In the setting without molecular-formula prompting and using combined ^13^C + ^1^H spectra as input, the model achieves 67.07% top-1 accuracy and 70.81% top-5 accuracy. This result further demonstrates that accurate structure elucidation can be achieved based solely on spectral information.

### Structure prediction on experimental 1D NMR dataset

#### Zero-shot transfer to experimental NMR data

We next evaluate zero-shot transfer of the proposed model from simulated training data to experimental NMR spectra, without any fine-tuning on real measurements. This setting is challenging due to the simulation–experiment domain gap arising from spectral distortions, experimental noise, and variations in acquisition conditions. For each spectrum, the autoregressive decoder generates up to 100 candidate structures using beam search and we report top-*k* accuracy for *k* = 1, 5, 10, and 100. The extended candidate list also provides the basis for the re-ranking analysis presented later.

Zero-shot results on experimental spectra are summarized in Table 2. Overall, the proposed model achieves performance comparable to the zero-shot baseline from Alberts *et al.*^21^ when evaluated on the chemical education dataset. However, a significant performance drop is observed relative to results on simulated test spectra, for example the top-1 accuracy with ^1^H input has dropped from 71.39% to 19.91%. To further analyze this effect, we partition the experimental dataset into two subsets: an overlap subset containing 96 molecules that are also present in the multimodal simulation dataset, and an unseen subset of 130 molecules that are entirely unseen during training. Comparing the overlap subset with simulated-data performance reveals a clear degradation across all input modalities: even for identical molecular structures, discrepancies in simulated and experimental spectra significantly reduce prediction accuracy. This degradation is particularly pronounced for ^1^H and combined ^13^C + ^1^H inputs, whereas ^13^C spectra are comparatively less affected. Proton spectra are typically more sensitive to experimental conditions and exhibit denser peak overlap, making simulated representations less transferable, whereas ^13^C spectra provide more stable structural cues. In addition, under strong domain mismatch, incorporating less transferable ^1^H features can introduce conflicting spectral evidence, which explains why the combined ^13^C + ^1^H setting does not initially outperform ^13^C alone.

**Table 2:** Zero-shot transfer and fine-tuning performance on the experimental Chemical Education dataset. Top-*k* structure prediction accuracy (%) is reported for overlap and unseen molecular subsets under different spectral input configurations. External baseline results from prior work^21^ are shown for reference

Mode	Spectra input	Test set	Top-1	Top-5	Top-10	Top-100
Alberts^21^ zero-shot baseline	H	Full	20.00	31.58	—	—
	C	Full	48.42	66.32	—	—
Zero-shot	H	Overlap	17.71	34.38	41.67	53.12
		Unseen	21.54	30.77	36.92	45.38
	C	Overlap	57.29	70.83	80.21	88.54
		Unseen	41.54	62.31	68.46	77.69
	C + H	Overlap	32.29	53.12	59.38	67.71
		Unseen	33.08	45.38	49.23	55.38
Fine-tune	H	Unseen	55.38	76.15	80.77	90.00
	C	Unseen	53.08	80.77	84.62	92.31
	C + H	Unseen	66.15	81.54	86.15	90.77

Interestingly, the difference in accuracy between the overlap and unseen subsets is modest for all modalities. This observation suggests that simulation–experiment spectral domain shift is a major contributor to the zero-shot performance loss. Because molecular novelty and structural complexity may also affect prediction difficulty, we further compared the overlap and unseen subsets using molecular-size, ring-system, and fingerprint-based chemical-space descriptors in the SI (Section S12). These analyses indicate that the overlap and unseen subsets are broadly comparable in molecular complexity, supporting the interpretation that spectral domain shift plays a central role in the observed zero-shot degradation.

#### Fine-tune performance

To mitigate the simulation–experiment domain gap, we further fine-tune the model using the overlap subset of experimental data and evaluate performance on the held-out unseen subset. This setting isolates the effect of adapting to experimental spectral characteristics while preserving a strict test on previously unseen molecular structures. For completeness, cross-validation results on the full experimental dataset and detailed comparisons with prior work are provided in the SI (Section S6).Fine-tuning leads to significant performance improvements across all spectral modalities as shown in Table 2. Notably, training on only around 100 experimental spectrum–molecule pairs is sufficient to recover a large fraction of the accuracy loss observed in the zero-shot setting, bringing performance closer to that achieved on simulated test data. The gains are particularly pronounced for ^1^H and combined ^13^C + ^1^H inputs, indicating that these modalities benefit most from adapting to experimental characteristics. The best-performing multimodal ^13^C + ^1^H configuration after fine-tuning indicates the advantage of integrating complementary spectral information once the modality-specific representations are better aligned with experimental data.

### Re-ranking based on chemical shift prediction

**Fig. 4:**
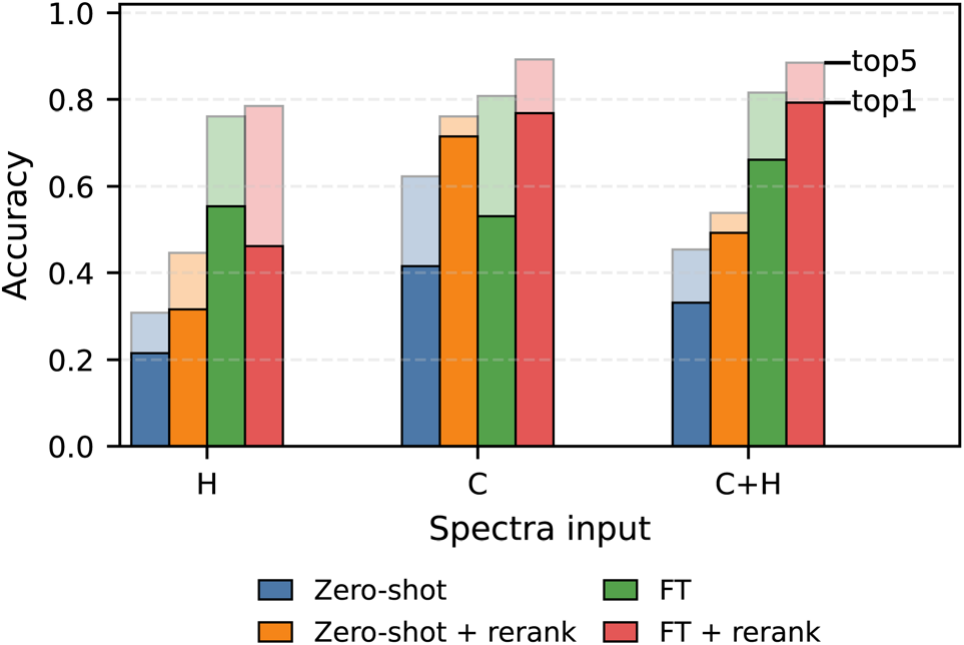
Effect of chemical-shift-based re-ranking on experimental structure prediction accuracy. Top-1 and top-5 accuracy on the Chemical Education dataset are shown for zero-shot prediction and after fine-tuning, with and without re-ranking, across ^1^H-only, ^13^C-only, and combined inputs. The predictions from combined input are re-ranked with only the ^13^C spectra. Re-ranking consistently improves top-rank retrieval quality, particularly in the zero-shot regime, highlighting the benefit of integrating external spectral agreement scoring with generative Transformer models. Full data table is included in the SI (Section S7).

We next evaluate the impact of chemical-shift-based re-ranking on structure prediction accuracy across different spectral inputs (Fig. 4). We note that re-ranking does not introduce new candidates but only reorders the generated list. Therefore, its effectiveness depends on the presence of the correct structure within the candidate pool and cannot recover correct structures that are absent from the top-*k* predictions. Re-ranking consistently improves both top-1 and top-5 accuracy in the zero-shot setting for all three input configurations. For ^13^C inputs, zero-shot prediction followed by re-ranking even outperforms the corresponding fine-tuned model. These results demonstrate that re-ranking serves as a practical mechanism that can improve top-rank retrieval quality without modifying the underlying generative model.

In contrast, improvements after re-ranking are generally smaller in the fine-tuned setting. This might be the saturation effect that reflects the already strong ranking quality achieved by fine-tuning, which leaves limited room for further refinement through external scoring. We also observe that re-ranking based on ^1^H chemical shifts provides the least improvement among the evaluated modalities. Compared with ^13^C, proton chemical shifts exhibit greater sensitivity to experimental conditions, which may reduce the discriminative power of shift-based agreement metrics. This trend is further supported by multimodal experiments. In addition to re-ranking with ^13^C, we also tried to ^1^H-based re-ranking to predictions from the combined ^13^C + ^1^H model. In this case, the zero-shot top-1 accuracy decreased from 49.23% to 35.38%, suggesting that incorporating proton shifts into the re-ranking stage is still challenging. Future work will therefore focus on developing more effective re-ranking strategies for jointly leveraging both carbon and proton spectral information.

**Fig. 5:**
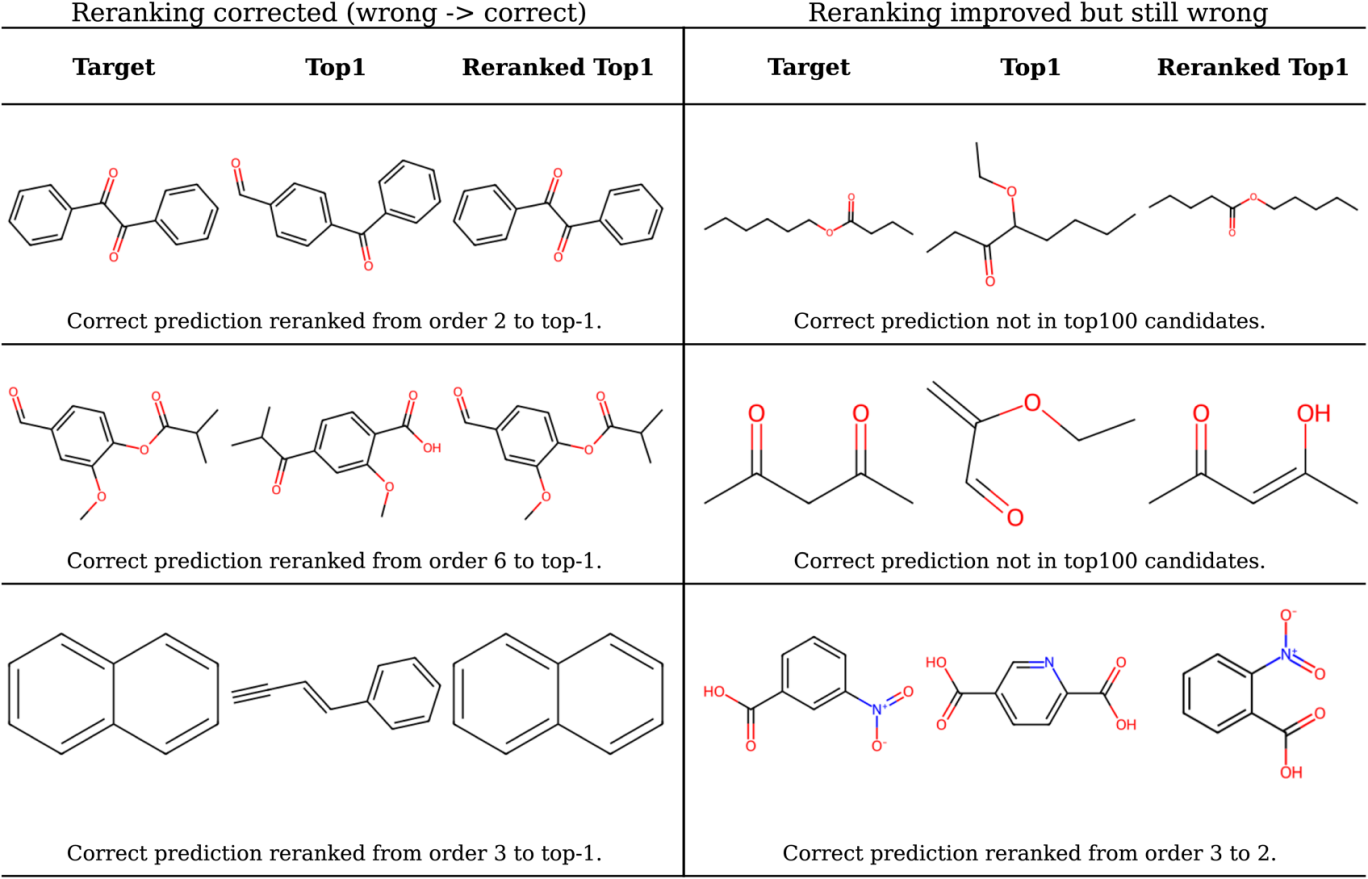
Representative qualitative examples illustrating the impact of chemical-shift-based re-ranking. Left: cases where re-ranking corrects an initially incorrect top-1 predictions. Right: cases where re-ranking imperfectly improves structural similarity to the target, including examples involving substructural corrections and tautomeric alternatives. These results demonstrate that re-ranking enhances both quantitative accuracy and chemical interpretability of model predictions.

To further provide qualitative insight into the re-ranking behavior, Fig. 5 shows representative prediction cases before and after chemical-shift-based re-ordering. In the corrected examples on the left, re-ranking successfully promotes the ground-truth structure to the top position by identifying more consistent functional-group arrangements. In these cases, the initial top-1 predictions often contain subtle but chemically important errors, such as misplaced carbonyl groups or incorrect substitution patterns on aromatic rings, which are resolved after incorporating chemical-shift agreement.

The examples on the right highlight situations where re-ranking improves the prediction but does not fully recover the exact target structure. For the 1st and 2nd examples, the correct molecule is not in the top-100 candidates. In these cases, the re-ranked top-1 predictions move closer to the correct molecular framework by recovering key substructural features, indicating that the scoring function provides meaningful chemical guidance. In the 3rd example, the target structure is present within the candidate pool. Although re-ranking does not elevate it to the top-1 position, it is ranked highly (second overall), indicating that the scoring step remains effective in prioritizing chemically consistent candidates. Besides, for the second example the re-ranked top-1 prediction corresponds to a tautomeric form of the target molecule, which remains challenging to distinguish based solely on NMR-derived evidence. Given the strong coupling between NMR observables and tautomeric equilibria, one promising future direction is to use the spectrum-based generator to propose candidate structures while employing additional physicochemical or thermodynamic scoring methods to further resolve tautomeric states.^54,55^ Together, these qualitative cases demonstrate that re-ranking not only increases quantitative accuracy but also enhances the chemical interpretability and practical utility of spectrum-to-structure predictions.

### Performance on experimental HSQC dataset

To further evaluate the ability of our model to utilize HSQC information for molecular structure prediction, we assess performance on the experimental HMDB-HSQC dataset. This small benchmark contains 74 metabolites, of which 50 have structural overlap with the multimodal simulated training dataset and 24 are previously unseen. Compared with the Chemical Education dataset, the HMDB molecules are generally larger and exhibit increased structural complexity, providing a more challenging test. We note that HSQC spectra in this benchmark are reconstructed from processed cross-peak lists rather than obtained as fully digitized raw spectral matrices. Consequently, fine spectral characteristics such as peak shape and experimental noise are not explicitly represented, which may influence prediction difficulty and model generalization. Future work will focus on learning directly from raw experimental 2D NMR data as such datasets become available.

**Fig. 6:**
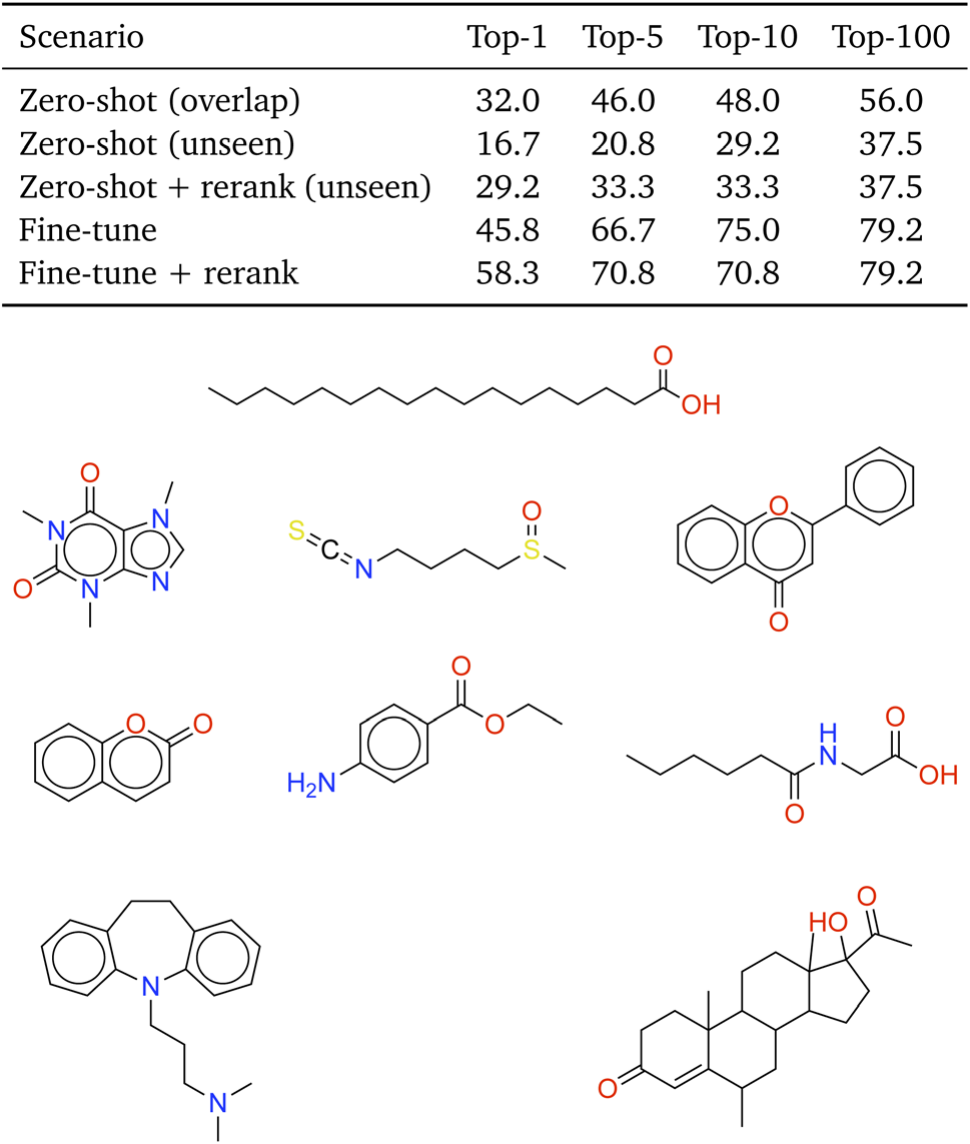
Structure prediction performance and representative reranking examples on the experimental HMDB-HSQC dataset. Top: top-*k* accuracy for zero-shot prediction on the overlap and unseen subsets, as well as after fine-tuning and chemical-shift-based reranking. Bottom: selected examples of correctly predicted molecules.

We report zero-shot performance separately for the overlap and unseen subsets in the table of Fig. 6. On the overlap subset, the model achieves a moderate top-1 accuracy of 32.0%, substantially lower than the 67.97% observed on the simulated multimodal test set, highlighting a persistent simulation–experiment domain gap. This reduction occurs despite identical underlying molecular structures, indicating that discrepancies between simulated and experimental HSQC characteristics constitute a major source of performance degradation. Performance further decreases on the unseen subset, reflecting the additional challenge of generalizing to larger and more structurally complex molecules. Fine-tuning on the small overlap dataset improves the top-1 accuracy on unseen molecules from 16.7% to 45.8%, demonstrating that even limited experimental adaptation can substantially reduce the sim–exp discrepancy and bring performance closer to that observed on simulated data. In addition, chemical-shift-based reranking consistently improves both zero-shot and fine-tuned predictions, with the combination of fine-tuning and reranking yielding the highest overall accuracy on this benchmark. Further analysis of the re-ranking influence on the correct prediction order are provided in the SI (Section S9).

To further illustrate the qualitative performance of the NMRViT model on HSQC-driven structure prediction, Fig. 6 presents representative examples of correctly predicted molecules in HMDB-HSQC dataset (full prediction list for each molecule is included in SI Section S10). Compared with the examples discussed for the Chemical Education dataset, these HSQC cases are generally more challenging due to the increased structural complexity. The correctly predicted examples span chemically diverse scaffolds, including conjugated aromatic systems, long aliphatic chains, and rigid polycyclic frameworks. Together with the quantitative improvements summarized in the top table of Fig. 6, these results demonstrate that the NMRViT model pretrained on simulated HSQC data can be effectively transferred to experimental HSQC spectra and achieve reliable structure prediction when leveraging fine-tuning and re-ranking. More generally, the proposed model design and transfer strategy are expected to extend to other two-dimensional NMR modalities including COSY and HMBC as appropriate simulated and experimental datasets become available.

## Conclusions

In this work, we present an end-to-end Transformer framework for NMR-based molecular structure elucidation that unifies ^1^H, ^13^C, and HSQC NMR within a ViT-based encoder–decoder architecture. By operating directly on full 1D and 2D spectral intensity fields, the model eliminates reliance on peak picking and symbolic encodings. NMRViT achieves state-of-the-art performance on large-scale simulated benchmarks, and demonstrates encouraging transferability on experimental datasets after fine-tuning on limited samples. Furthermore, a chemical-shift-based re-ranking strategy consistently improves candidate selection, highlighting the benefit of integrating external scoring with data-driven generation for practical spectrum-to-structure applications. Looking forward, an important direction is to further enhance the robustness of NMR image-based Transformers to noisy and heterogeneous real-world spectral images, enabling the effective utilization of the vast amount of NMR data reported in the literature. Progress along this direction may also encourage the broader sharing of high-quality spectral measurements, thereby facilitating the development of spectrum-image-driven machine learning approaches for automated molecular structure elucidation. Such advances could enable NMR analysis to function as an autonomous analytical module within closed-loop, AI-guided robotic laboratories, where synthesis, characterization, and structural decision-making are seamlessly integrated into end-to-end discovery workflows.^56,57^

## Author contributions

Prof. Yingkai Zhang initiated and supervised the project. Chao Han developed the deep learning models, curated and processed the datasets, and conducted the computational experiments and data analysis. Dr Xiaolin Pan contributed to the curation of the experimental datasets and assisted in drafting the manuscript.

## Conflicts of interest

The authors declare no competing financial interest.

## Supplementary Material

SC-OLF-D6SC02352E-s001

## Data Availability

All datasets and codes used in this work are available. Source codes for data set preparation, model training, and model usage are at https://github.com/ChaoNyu/NMRViT. The trained models are available at https://doi.org/10.5281/zenodo.19153901. Supplementary information (SI) is available. See DOI: https://doi.org/10.1039/d6sc02352e.
