# Supplementary material for: End-to-end molecular structure elucidation from multimodal NMR spectra images using vision transformers
Source: Chem Sci. 2026 May 26;17(26):13072–82. doi: 10.1039/d6sc02352e (PMC13211313; doi:10.1039/d6sc02352e)
Supplement: SC-017-D6SC02352E-s001 [file SC-017-D6SC02352E-s001.pdf]

Supplementary Information (SI) for Chemical Science.
This journal is © The Royal Society of Chemistry 2026

Supporting Information
End-to-end Molecular Structure Elucidation from Multimodal NMR Spectra Images using Vision Transformers

Chao Han, Xiaolin Pan, and Yingkai Zhang

Contents

S1 Model Architecture and training details **2**

S2 Choice of patch dropout rate and patch size **3**

S3 Full table of ablation study on Multimodal Spectroscopic Dataset **4**

S4 NMR spectra sparsity analysis **5**

S5 Multimodal setting: comparison of early and late fusion performance **6**

S6 Cross-validation performance on Chemical Education Dataset **7**

S7 Full results of model performance on Chemical Education Dataset **8**

S8 Scoring function design for re-ranking methods **9**

S9 Re-ranking influence on correct predictions **11**

S10 Full reranking results for HMDB-HSQC dataset **13**

S11 Qualitative analysis of modality contributions on Multimodal dataset **15**

S12 Structural-complexity and chemical-space comparison of overlap and unseen experimental subsets **16**

S1 Model Architecture and training details

The NMRViT framework adopts an encoder–decoder Transformer architecture for spectrum-to-structure prediction, as summarized in the main text. In practical implementation, spectral inputs are converted to token sequences using convolution-based patch embedding layers. A learnable classification token is prepended to each input sequence and trained jointly with the patch tokens. The complete encoder token sequence is provided to the decoder through cross-attention without intermediate pooling.

Patch-dropout regularization is applied during training by randomly removing a subset of patch tokens while retaining the classification token, improving robustness to sparse spectral inputs. The molecular formula is provided as a prefix in the decoder input sequence and is excluded from loss computation. SMILES strings are tokenized using a chemistry-aware scheme following previous sequence-to-molecule models[3]. All models share a common configuration with hidden dimension 512, 8 attention heads, and 6 encoder and decoder layers. The training loss is token-level cross-entropy with label smoothing at 0.1. Training is implemented using PyTorch Lightning and executed on the single NVIDIA L40S GPU. Typical full-training runs for spectrum-to-SMILES prediction require 25-30 hours depending on spectral modality. We summarize the hyperparameters in the table S1. Further discussion on the choice of patch size and patch dropout rate is in section S2.

Table S1: Hyperparameter list of the NMRViT models (single modality).

**Parameter**	**Value**
Hidden dimension	512
Number of attention heads	8
Encoder layers	6
Decoder layers	6
Feedforward dimension	2048
Dropout rate	0.1
Vocabulary size	60
Maximum decoder length	100 tokens
Label smoothing	0.1
Optimizer	AdamW
Learning rate	3×10^{-4}
Weight decay	0.01
Warmup steps	10 000
Learning rate schedule	Cosine decay
Gradient clipping	1.0
Batch size	512
Training epochs	200

S2 Choice of patch dropout rate and patch size

While the model design adopts a standard encoder–decoder Transformer architecture with commonly-used hyperparameter settings, the patch-dropout rate and the patch size require systematic exploration to identify an optimal combination. The patch size determines the granularity at which spectral signals are tokenized, and the patch-dropout rate controls the strength of regularization by randomly masking spectral regions during training.

We performed a grid search over patch size and patch-dropout rate for each spectral modality. To reduce the computational cost of repeated model training, hyperparameter tuning was conducted on a randomly selected subset with 10% of the full training set. Model selection was based on the best validation accuracy evaluated on the same validation split used in the main experiments. For 1D spectra, we examined the patch sizes of [25, 50, 100] and patch dropout rate of [0, 0.25, 0.5]. For 2D HSQC spectra, square patch sizes of 32, 64, and 128 were evaluated. We did not further reduce the patch size as a smaller patch size such as 16×16 would increase the token sequence length from 256 to 1024, resulting in substantially higher computational cost and potential overfitting.

The accuracy is shown in Table S2. For 1D spectra the combination of patch size=50 and patch dropout rate=0.25 achieved the highest validation accuracy. On the 2D HSQC spectra, the smallest patch size at 32 is most beneficial for capturing localized cross-peak patterns. As observed for the 1D modalities, a moderate patch-dropout rate of 0.25 provided the best overall performance. Based on these results, the final model configuration adopts a unified patch-dropout rate of 0.25 for all spectral inputs, with patch size set to 50 for 1D spectra and 32 for HSQC.

Table S2: Validation accuracy (%) for different patch sizes and patch-dropout rates in the hyperparameter sweep.

Modality	Dropout	Patch size		
		Small	Medium	Large
^{1}H	0	12.38	12.51	13.17
	0.25	16.02	**17.47**	16.46
	0.5	16.14	17.18	15.90
^{13}C	0	3.85	3.86	4.49
	0.25	5.08	**5.31**	5.00
	0.5	5.22	5.08	4.44
HSQC	0	11.14	8.36	6.59
	0.25	**12.41**	9.69	7.07
	0.5	10.45	8.48	5.63

Small/Medium/Large patch sizes correspond to 25/50/100 for 1D spectra and 32/64/128 for HSQC.

S3 Full table of ablation study on Multimodal Spectroscopic Dataset

The Table S3 provides the full data of the Figure 3 in the main text.

Table S3: Full Ablation analysis on Multimodal Spectroscopic Dataset for different settings (% accuracy). MF means molecular formula prompt, PD means patch dropout. Each entry reports Top-1 / Top-5 accuracy.

**Input**	**w/o MF**	**w/o PD**	**Full model**
^{1}H	58.26 / 63.56	69.57 / 81.47	71.39 / 83.79
^{13}C	41.28 / 49.74	48.40 / 61.89	51.34 / 65.89
HSQC	51.06 / 59.23	65.56 / 77.98	67.97 / 81.59
^{13}C+^{1}H	67.07 / 70.81	70.28 / 81.67	74.07 / 85.68

S4 NMR spectra sparsity analysis

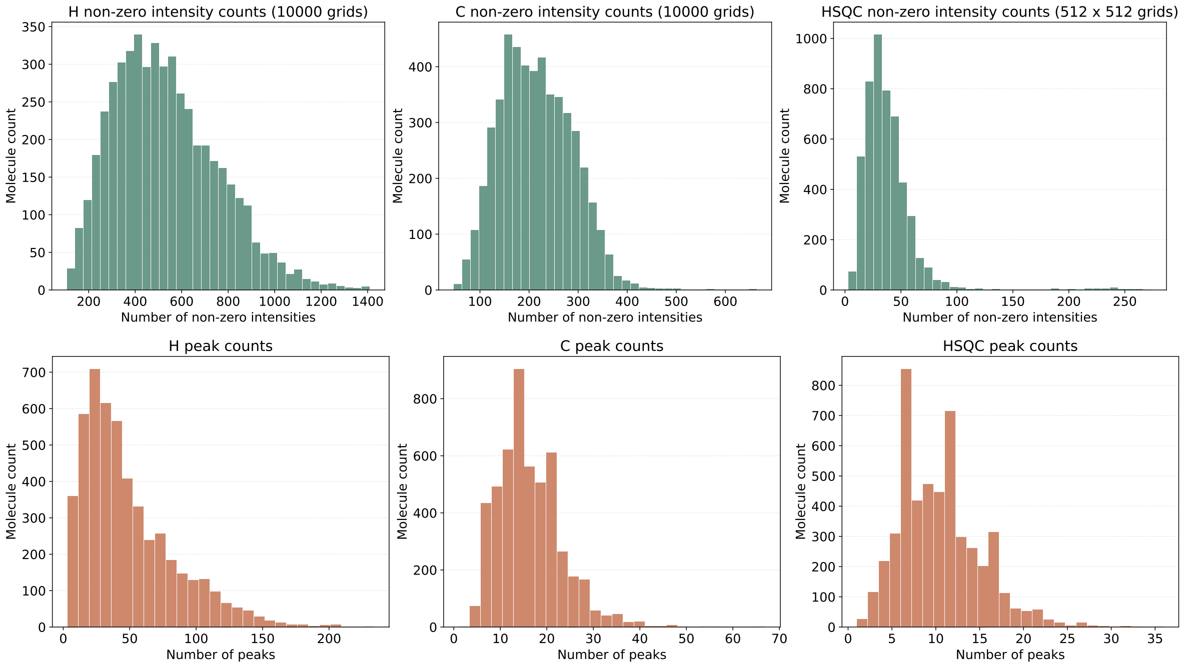

Figure S1: Statistical distributions of spectral sparsity and peak counts in the sampled Multimodal Spectroscopic Dataset. Top row: histograms of the number of non-zero intensity grid points for ^{1}H, ^{13}C and HSQC spectra. Bottom row: corresponding distributions of detected peak counts for each modality.

To characterize the statistical properties of spectral inputs in the simulated multimodal dataset, we analyze spectral sparsity on uniform chemical-shift grids. In this dataset, 1D ^{1}H spectra are represented as vectors of length 10 000 covering 10 to -2 ppm (resolution 0.0012 ppm), while ^{13}C spectra use the same vector length over 230 to -20 ppm (resolution 0.025 ppm). Two-dimensional HSQC spectra are stored as 512×512 intensity matrices spanning the same chemical-shift ranges for ^{1}H and ^{13}C.

Spectral sparsity is first quantified by counting grid points with normalized intensity greater than 10^{-4}. In addition, we estimate the number of spectral peaks using a simple local-maximum criterion: in 1D spectra, peaks correspond to thresholded points not smaller than their immediate neighbors, whereas in HSQC spectra peaks are defined as local maxima within a 3×3 neighborhood. The resulting distributions (Figure S1) indicate that only a small fraction of grid points contain signal across all modalities, confirming the intrinsically sparse nature of the simulated NMR inputs. This characteristic supports the use of patch dropout regularization in the proposed Transformer encoder, which encourages the model to focus on informative spectral regions.

S5 Multimodal setting: comparison of early and late fusion performance

In addition to the multimodal integration strategy described in the main text, we also evaluated an alternative late-fusion architecture for comparison with the early-fusion design. In the early-fusion model, each modality is first converted into patch embeddings using modality-specific embedding layers, and the resulting token sequences are concatenated before being processed by a single Transformer encoder. A shared class token and positional embeddings are then added to the fused sequence, enabling the encoder to operate on the combined multimodal representation.

In contrast, the late-fusion model maintains separate processing streams for different modalities during the encoding stage. For example, ^{13}C and ^{1}H spectra are first transformed into patch tokens using independent embedding layers and are then processed by separate Transformer encoders. This design allows each branch to learn modality-specific representations prior to cross-modal interaction. The encoded token sequences are subsequently concatenated and passed through a lightweight cross-attention compressor that reduces the full multimodal sequence to a fixed number of learned summary tokens. This compressed representation is then provided to the shared decoder for SMILES generation.

We compare the two architectures using combined ^{13}C and ^{1}H spectra as input. In the late-fusion configuration, each modality uses a patch size of 50 for spectra of length 10000, resulting in 200 tokens per modality before fusion. After concatenation, the joint sequence is compressed to 200 tokens in total, ensuring a comparable decoder input length while preserving modality-specific encoding. This setup enables a direct architectural comparison between early and late fusion under matched multimodal input conditions.

As summarized in Table S4, the early-fusion architecture achieves slightly higher prediction accuracy than the late-fusion model across all Top-k metrics. This result suggests that allowing cross-modal interactions from the initial encoding stage is beneficial for integrating complementary structural information from ^{13}C and ^{1}H spectra. The late-fusion design may also suffer from overfitting due to the increased number of model parameters introduced by the additional modality-specific encoders. Nevertheless, the overall performance difference remains moderate, indicating that both fusion strategies are capable of effectively utilizing multimodal spectral inputs for structure prediction.

Table S4: Performance comparison of early- and late-fusion architectures for multimodal ^{13}C + ^{1}H inputs on the test set.

Fusion method	Top-1 acc (%)	Top-5 acc (%)	Top-10 acc (%)
Early fusion	74.07	85.68	87.89
Late fusion	71.59	84.79	87.42

S6 Cross-validation performance on Chemical Education Dataset

To enable direct comparison with prior spectrum-to-structure studies, we evaluate model performance on the Chemical Education dataset with the same cross validation setting as in previous study[1]. In the study by Alberts et al., this dataset is used for fine-tuning and evaluation of models pretrained on large simulated datasets. After filtering for modality completeness and element consistency with their simulated training set, the authors report a subset of 171 molecules for downstream experiments. A five-fold cross validation is conducted on the dataset, with performance reported as the mean and standard deviation across folds.

In the main text of the present work, we focus on a fine-tuning strategy based on overlap/unseen splits with respect to the simulated multimodal training set, which better reflects the practical deployment scenario of transferring models to new experimental molecules. To facilitate a fair comparison with previous studies, we additionally perform five-fold cross-validation on the Chemical Education dataset, and report the corresponding results in Table S5. Overall, the cross-validation results show that both single-modality models and multimodal models achieve strong predictive accuracy on this relatively small benchmark. Compared with the Alberts fine-tuned baselines, our models demonstrate improved accuracy, which better reflects the generative nature of sequence-to-structure prediction. In addition, the relatively small standard deviations across folds suggest stable model behaviour despite the limited dataset size.

We note that the exact preprocessing pipeline used in the previous study is not fully documented. Following our own data curation and filtering procedures, we obtained 226 molecules suitable for cross-validation, slightly exceeding the subset size reported previously. This difference likely arises from variations in spectrum availability and molecular filtering criteria.

Table S5: Cross-validation performance on the Chemical Education dataset. Values are reported as mean ± standard deviation across folds.

**Setting**	**Top-1**	**Top-5**	**Top-10**	**Top-50**	**Top-100**
^{13}C (Alberts FT baseline)	0.58 ± 0.11	0.86 ± 0.10	–	–	–
^{1}H (Alberts FT baseline)	0.46 ± 0.08	0.73 ± 0.04	–	–	–
^{1}H	0.70 ± 0.07	0.88 ± 0.01	0.90 ± 0.02	0.92 ± 0.03	0.93 ± 0.02
^{13}C	0.67 ± 0.06	0.86 ± 0.06	0.90 ± 0.05	0.94 ± 0.06	0.94 ± 0.06
^{13}C+^{1}H	0.79 ± 0.06	0.90 ± 0.05	0.91 ± 0.06	0.94 ± 0.03	0.95 ± 0.04

S7 Full results of model performance on Chemical Education Dataset

In this section we summarize the full results on Chemical Education Dataset in the Table S6 as the values used to plot the Figure 4 in the main text.

Table S6: Effect of re-ranking on structure prediction accuracy. Top-1 and Top-5 accuracy on the Chemical Education dataset are shown for zero-shot prediction and after fine-tuning, with and without re-ranking, across ^{1}H-only, ^{13}C-only, and combined inputs.

Spectra input	Scenario	Top-1	Top-5
^{1}H	Zero-shot	0.2154	0.3077
^{1}H	Zero-shot + rerank	0.3154	0.4462
^{1}H	FT	0.5538	0.7615
^{1}H	FT + rerank	0.4615	0.7846
^{13}C	Zero-shot	0.4154	0.6231
^{13}C	Zero-shot + rerank	0.7154	0.7615
^{13}C	FT	0.5308	0.8077
^{13}C	FT + rerank	0.7692	0.8923
^{13}C+^{1}H	Zero-shot	0.3308	0.4538
^{13}C+^{1}H	Zero-shot + rerank	0.4923	0.5385
^{13}C+^{1}H	FT	0.6615	0.8154
^{13}C+^{1}H	FT + rerank	0.7923	0.8846

S8 Scoring function design for re-ranking methods

Table S7: Comparison of re-ranking strategies on the zero-shot predictions for HMDB-HSQC dataset. Reported values are Top-k accuracies (%).

Method	Top-1	Top-5	Top-10	Top-100
Zero-shot	27.03	37.84	41.89	50.00
+ Formula filter	29.73	43.24	45.95	50.00
+ Cosine similarity	21.62	33.78	40.54	50.00
+ Local evidence score	32.43	44.59	45.95	50.00
+ Hungarian matching score	41.89	48.65	50.00	50.00

In addition to the NMRViT model that can predict molecular candidates with given NMR spectra, we further introduce the re-ranking methods as a post-processing step to provide additional chemistry-informed constraint beyond sequence likelihood from the Transformer. In our study we retain top-100 SMILES generated by NMRViT as the candidate pool. Zero-shot performance is evaluated by comparing the generated SMILES strings with the reference structures after conversion to non-stereo canonical SMILES, and reporting Top-k accuracy. To conduct the re-ranking for the predictions, the generated candidates are first subjected to a validity check and filtered according to the correct molecular formula. For the remaining molecules, atom-wise chemical shifts are predicted using the CSTShift model[2]. These predicted shifts are then compared with the experimental spectra to compute a spectral consistency score, which is used to reorder the candidate list.

Different score designs are considered for the re-ranking stage. In addition to the local-evidence score used in the main analysis, we evaluate a cosine similarity score and a peak-matching strategy based on Hungarian assignment. For the cosine similarity approach, predicted chemical shifts are reconstructed as stick-style spectra on the same discretized chemical-shift grid as the experimental spectrum. The similarity between the reconstructed and experimental spectra is then computed as the cosine similarity of their normalized intensity vectors.

To investigate the influence of peak-picking assumptions, we further compare a peak-list matching strategy. The HMDB-HSQC benchmark dataset provides experimental data in peak-list format, enabling direct comparison between predicted (^{13}C, ^{1}H) shift pairs and experimental cross-peaks. In this setting, predicted HSQC peak coordinates are reconstructed from CSTShift atom-level predictions by identifying bonded C–H pairs in the candidate molecule. A cost matrix is then constructed using normalized Euclidean distances in the two-dimensional chemical-shift space, and the optimal assignment between predicted and experimental peaks is obtained using the Hungarian algorithm. The final score is defined as the negative average matching cost with an additional penalty for unmatched peaks.

The comparison of different scoring strategies is summarized in Table S7. Applying only molecular-formula filtering leads to a modest improvement over the zero-shot baseline. The inferior perfor-

mance of the cosine-similarity score likely arises from the HSQC spectra sparsity analyzed in Section S4, where signal is confined to a few localized cross-peaks and large regions remain zero. In contrast, the local-evidence score provides a consistent improvement in Top-1 accuracy, demonstrating the importance of localized spectral agreement. The Hungarian peak-matching strategy yields the largest performance gain, increasing Top-1 accuracy to 41.9%. This result highlights the potential for further improvement in score design when working with unannotated spectra, where incorporating peak-region matching strategies may provide more discriminative structural evidence. It also suggests that peak-assignment–based approaches offer a practical alternative for reranking when experimental peak lists are available.

S9 Re-ranking influence on correct predictions

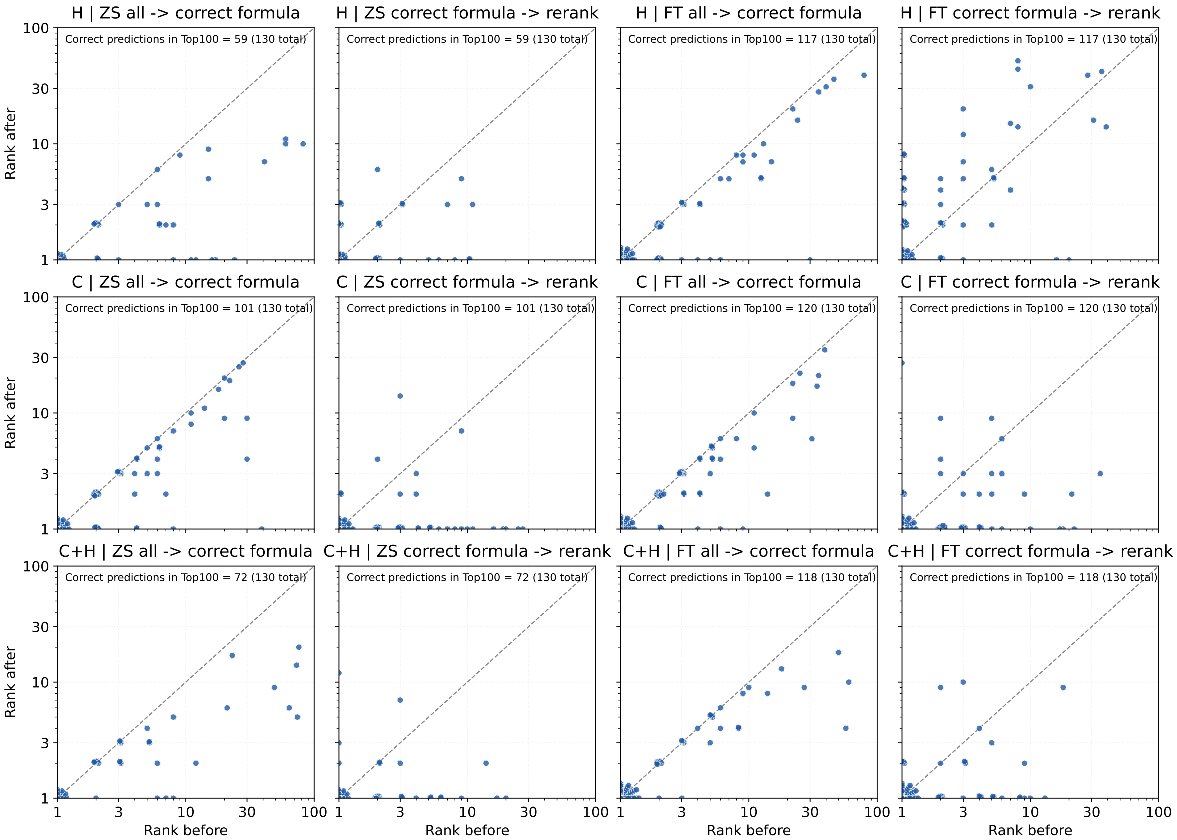

Figure S2: Order before/after re-ranking for molecules that are predicted correctly among top 100 candidates on Chemical Educational Dataset (unseen subset). Each panel plots the rank before the step on the x-axis and the rank after the step on the y-axis, using logarithmic scales from 1 to 100, with the dashed diagonal indicating no rank change.

In this section we show the re-ranking influence on the correct predictions by comparing their order before and after re-ranking. We evaluate this on the unseen subset of Chemical Educational Dataset for both zero-shot and fine-tuned model predictions, where we track molecules whose correct target appears within the top 100 candidates. Since the sanity check and the molecular formula filter will also change the order, we decompose the reranking into two parts: formula filter and score reranking. For each input setting (^{1}H, ^{13}C, and ^{13}C+^{1}H) and for both zero-shot (ZS) and fine-tuned (FT) models, we compare the rank of the correct prediction across two transitions: from the candidate list to the formula-filtered list, and from the formula-filtered list to the re-ranked list.

The comparison is shown in Figure S2. The molecular-formula filtering step generally improves the ranking of correct structures by removing chemically invalid or formula-inconsistent candidates, thereby sharpening the candidate list prior to score-based re-ordering. For zero-shot predictions, the subsequent re-ranking shows a clearly beneficial effect. Most points fall in the lower triangular region, and many accumulate along the bottom boundary, indicating that correct candidates are

often promoted to higher ranks or even selected as top-1. In contrast, the impact on fine-tuned predictions is more mixed, particularly for the ^{1}H and ^{13}C+^{1}H settings, where more cases exhibit rank deterioration. Nevertheless, these changes are typically confined to the lower-left region of the plots, implying that the correct structures generally remain within the top-10 predictions and that overall retrieval quality is not substantially compromised.

S10 Full reranking results for HMDB-HSQC dataset

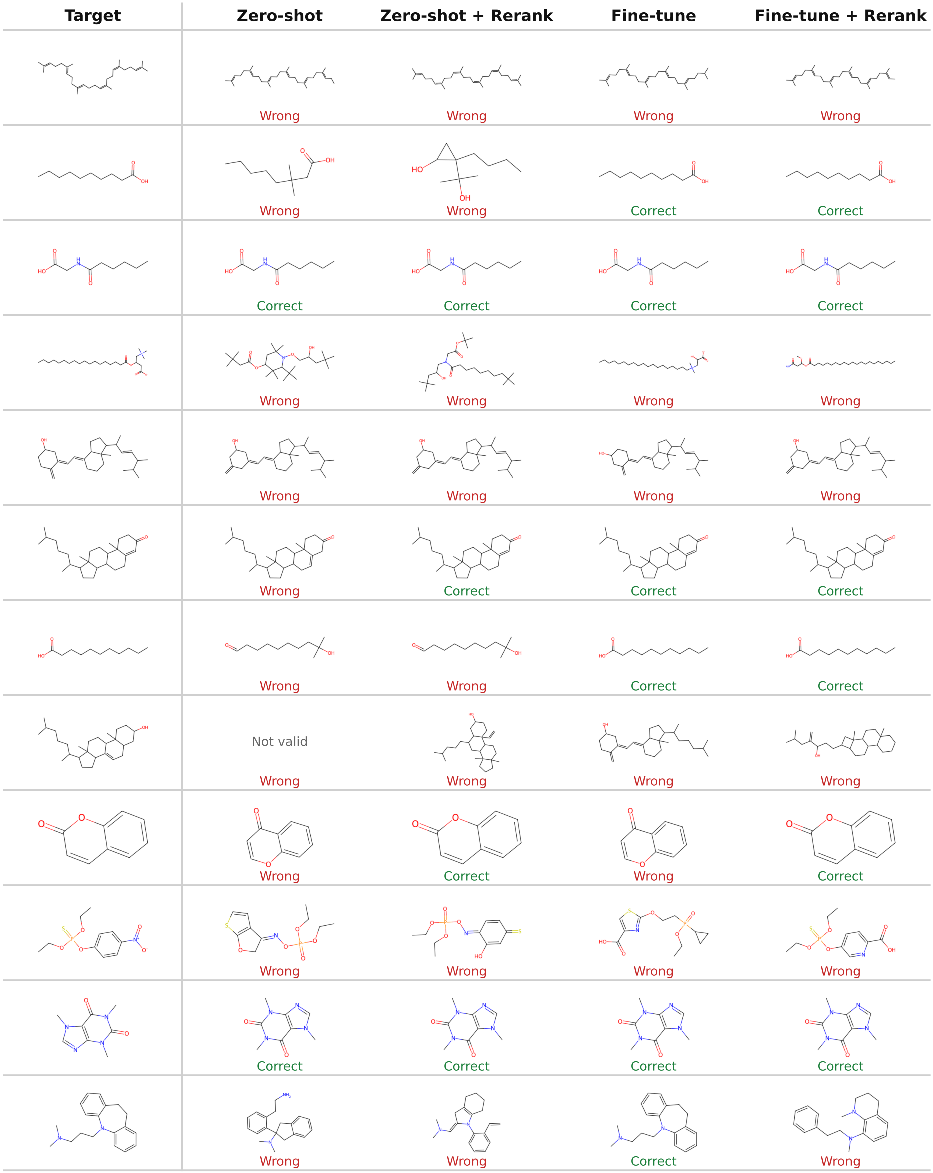

Figure S3: Top-1 molecule predictions on the HMDB-HSQC test set, page 1 of 2. Columns show the target, zero-shot, zero-shot with reranking, fine-tuned, and fine-tuned with reranking results. Correct predictions are labeled in green and incorrect predictions in red.

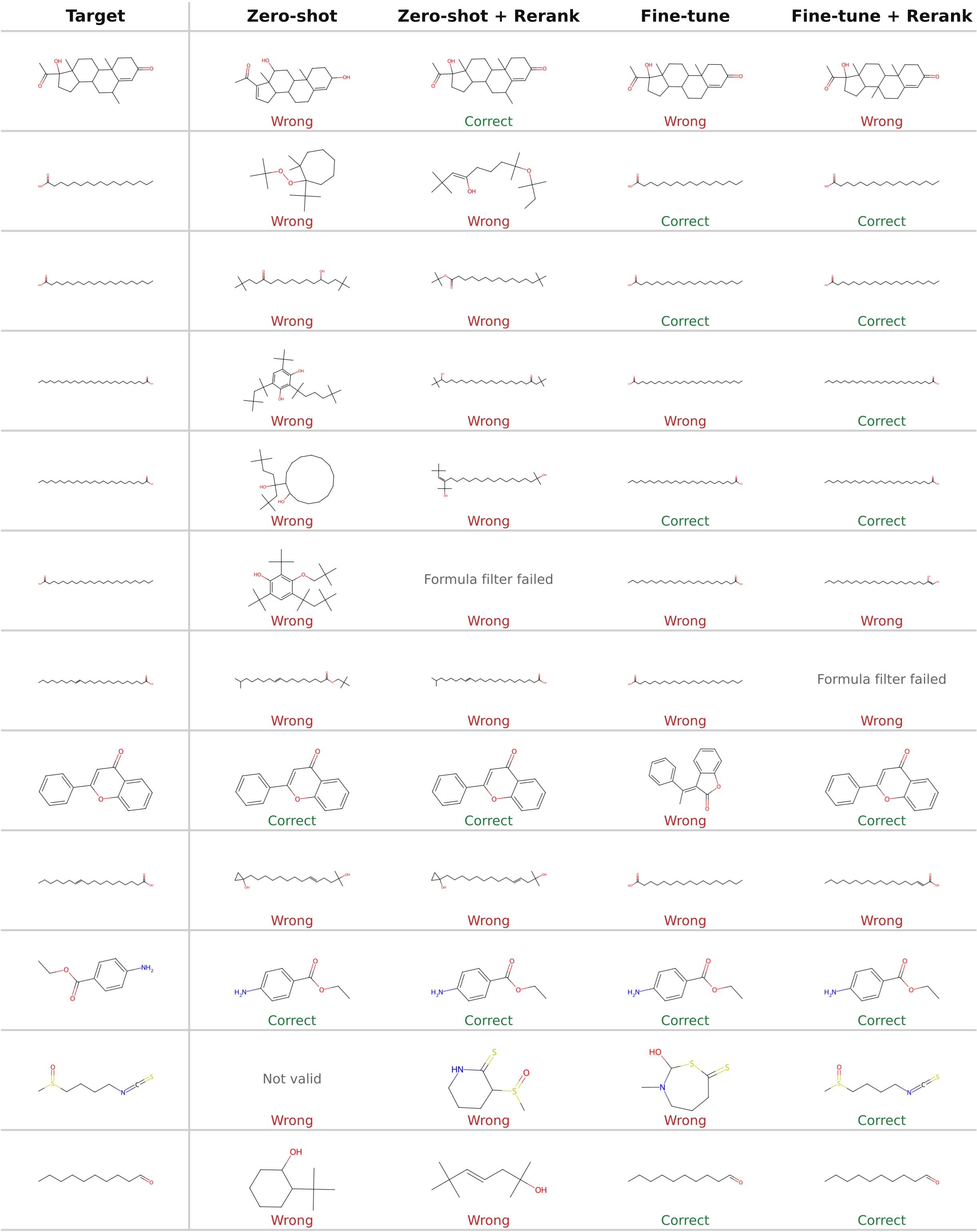

Figure S4: Top-1 molecule predictions on the HMDB-HSQC test set, page 2 of 2.

S11 Qualitative analysis of modality contributions on Multimodal dataset

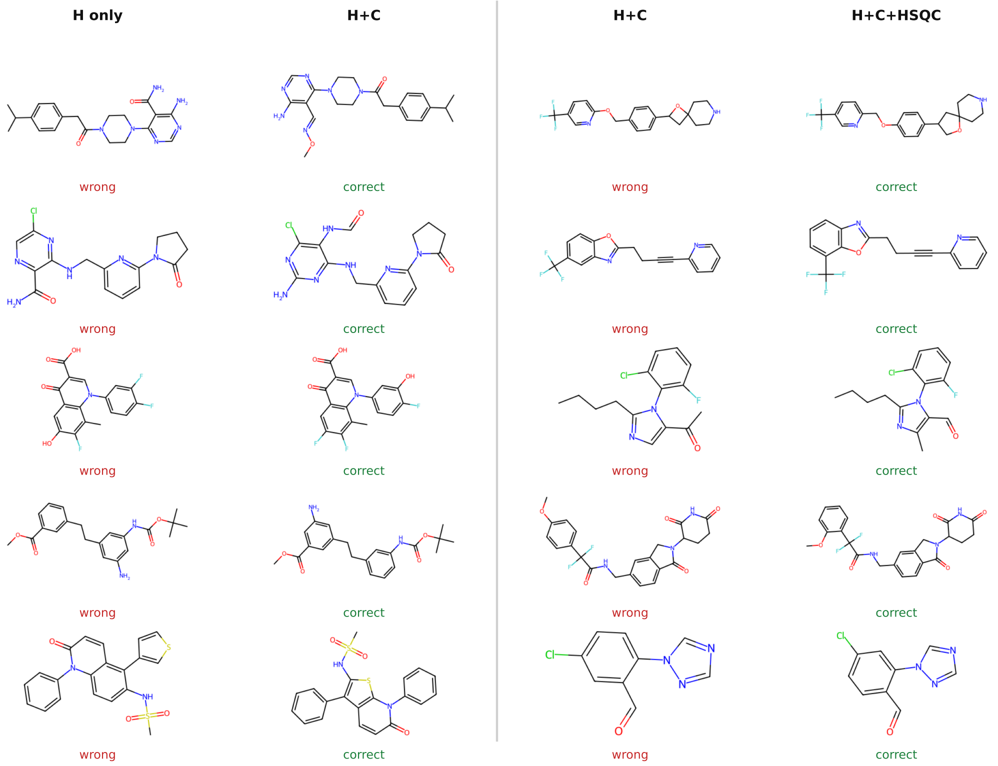

Figure S5: Representative examples illustrating modality-dependent correction of structure predictions. Left: examples where the ^{1}H-only model gives an incorrect top-1 prediction, while the combined 1H+^{13}C model gives the correct structure. Right: examples where the ^{1}H+^{13}C model gives an incorrect top-1 prediction, while the ^{1}H+^{13}C+HSQC model gives the correct structure.

To provide additional insight into how different NMR modalities contribute to structure prediction, we examined representative cases in which adding complementary spectral inputs changes an incorrect prediction into a correct one. As shown in Fig. S5, several molecules that are incorrectly predicted by the ^{1}H-only model are correctly recovered when ^{13}C NMR is added, indicating that carbon chemical-shift information provides complementary constraints on molecular constitution and helps resolve ambiguities in proton spectra. We also compare predictions from the ^{1}H+^{13}C model with those from the ^{1}H+^{13}C +HSQC model. In these examples, adding HSQC information corrects errors that remain after using both 1D modalities, consistent with the role of HSQC in providing direct correlations between proton and carbon environments. The corrected cases include changes in functional-group placement, heteroatom arrangement, aromatic substitution patterns, and scaffold connectivity. These examples suggest that the performance gains from multimodal inputs arise from chemically meaningful complementary information rather than only from increased model capacity.

S12 Structural-complexity and chemical-space comparison of overlap and unseen experimental subsets

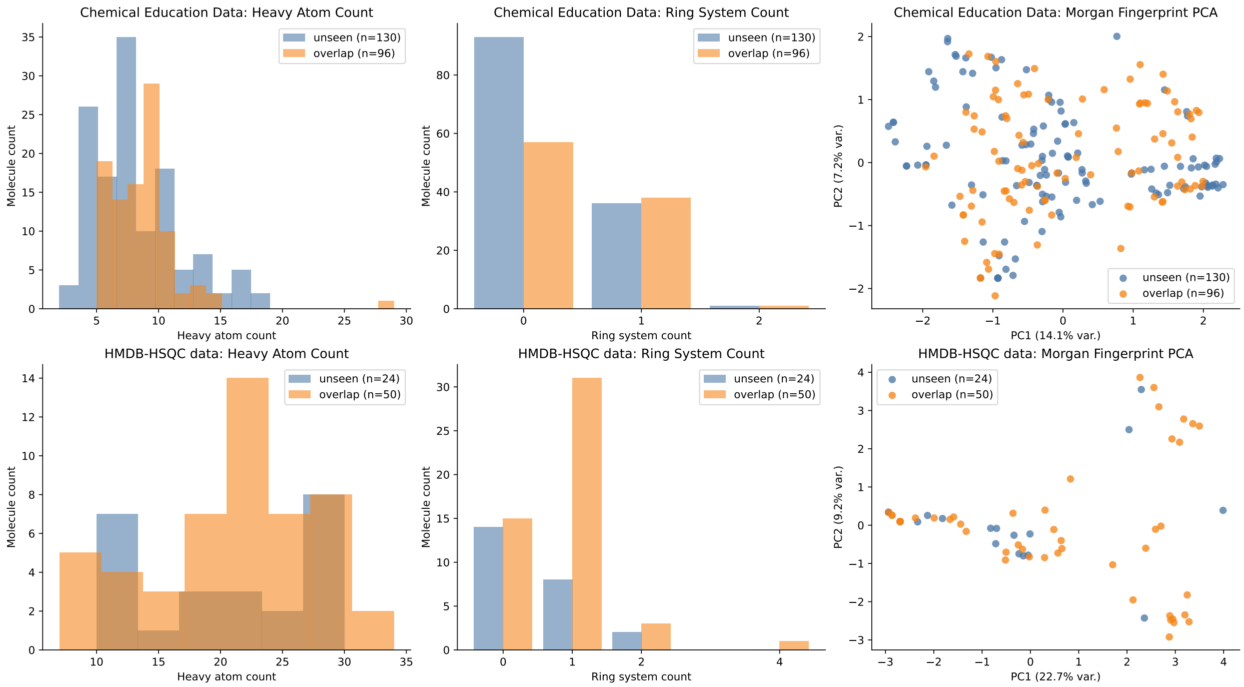

Figure S6: Structural-complexity and chemical-space comparison between overlap and unseen experimental subsets. Heavy-atom-count distributions, Morgan fingerprint PCA projections, and ring-system-count distributions are shown for the Chemical Education dataset and the HMDB-HSQC dataset. The overlap subset contains molecules whose structures are also present in the simulated training dataset, whereas the unseen subset contains molecules absent from the simulated training set.

In the experimental evaluations, we used overlap and unseen subsets to illustrate the effect of simulation–experiment spectral domain shift on model transferred performance. The substantial performance degradation observed on the overlap subsets, where the molecular structures are present in the simulated training set but the spectra are experimentally acquired, indicates that the simulation–experiment gap strongly affects zero-shot transfer. Here, we further analyze the molecular characteristics of the overlap and unseen subsets to ensure that the prediction tasks on these subsets are not substantially confounded by differences in molecular structural complexity. As shown in Fig. S6 , the heavy-atom-count and ring-system-count distributions are similar between the overlap and unseen subsets for both the Chemical Education dataset and the HMDB-HSQC dataset. The molecular-size and ring-system distributions of the overlap subsets broadly cover those of the unseen subsets, indicating that the unseen molecules are not systematically larger or more ring-rich. To further compare the overall chemical space, we computed Morgan fingerprints for all molecules and used principal component analysis (PCA) to visualize their distributions. The PCA projections show that the overlap and unseen subsets are extensively intermingled in finger-

print space, rather than forming clearly separated clusters. This analysis supports that the overlap and unseen subsets are broadly comparable in molecular complexity, strengthening the interpretation that the observed zero-shot degradation is mainly associated with the simulation–experiment spectral domain gap rather than a systematic increase in molecular difficulty in the unseen subsets.

References

[1] Marvin Alberts, Nina Hartrampf, and Teodoro Laino. Automated Structure Elucidation at Human-Level Accuracy via a Multimodal Multitask Language Model. ChemRxiv, 2025. preprint.

[2] Chao Han, Dongdong Zhang, Song Xia, and Yingkai Zhang. Accurate Prediction of NMR Chemical Shifts: Integrating DFT Calculations with Three-Dimensional Graph Neural Networks. *Journal of Chemical Theory and Computation*, 20(12):5250–5258, June 2024.

[3] Philippe Schwaller, Teodoro Laino, Théophile Gaudin, Peter Bolgar, Christopher A. Hunter, Costas Bekas, and Alpha A. Lee. Molecular Transformer: A Model for Uncertainty-Calibrated Chemical Reaction Prediction. *ACS Central Science*, 5(9):1572–1583, September 2019.
